# Filmless quality assurance of a Leksell Gamma Knife® Icon™

**DOI:** 10.1002/acm2.13070

**Published:** 2020-12-10

**Authors:** Borna Maraghechi, Taeho Kim, Timothy J. Mitchell, S. Murty Goddu, Joe Dise, James A. Kavanaugh, Jacqueline E. Zoberi, Sasa Mutic, Nels C. Knutson

**Affiliations:** ^1^ Departments of Radiation Oncology Washington University in St. Louis St. Louis MO USA

**Keywords:** film dosimetry, Gamma Knife^®^ Icon™, quality assurance, radiation profile, small field dosimetry

## Abstract

**Purpose:**

The annual quality assurance (QA) of Leksell Gamma Knife^®^ (LGK) systems are typically performed using films. Film is a good candidate for small field dosimetry due to its high spatial resolution and availability. However, there are multiple challenges with using film; film does not provide real‐time measurement and requires batch‐specific calibration. Our findings show that active detector‐based QA can simplify the procedure and save time without loss of accuracy.

**Methods:**

Annual QA tests for a LGK Icon™ system were performed using both film‐based and filmless techniques. Output calibration, relative output factors (ROF), radiation profiles, sector uniformity/source counting, and verification of the unit center point (UCP) and radiation focal point (RFP) coincidence tests were performed. Radiochromic films, two ionization chambers, and a synthetic diamond detector were used for the measurements. Results were compared and verified with the treatment planning system (TPS).

**Results:**

The measured dose rate of the LGK Icon was within 0.4% of the TPS value set at the time of commissioning using an ionization chamber. ROF for the 8 and 4‐mm collimators were found to be 0.3% and 1.8% different from TPS values using the MicroDiamond detector and 2.6% and 1.9% different for film, respectively. Excellent agreement was found between TPS and measured dose profiles using the MicroDiamond detector which was within 1%/1 mm vs 2%/1 mm for film. Sector uniformity was found to be within 1% for all eight sectors measured using an ionization chamber. Verification of UCP and RFP coincidence using the MicroDiamond detector and pinprick film test was within 0.3 mm at isocenter for both.

**Conclusion:**

The annual QA of a LGK Icon was successfully performed by employing filmless techniques. Comparable results were obtained using radiochromic films. Utilizing active detectors instead of films simplifies the QA process and saves time without loss of accuracy.

## 1 INTRODUCTION

The Leksell Gamma Knife^®^ (LGK) Icon™ (Elekta A.B., Stockholm, Sweden) is a specialized intracranial stereotactic radiosurgery system. Gamma Knife radiosurgery can be used to treat brain tumors, arteriovenous malformations, and several neurological conditions such as trigeminal neuralgia and essential tremors in a single fraction or multiple fractions.^1^ For these treatments, the patient’s head is immobilized using either a rigid frame or thermoplastic facemask.^2^, ^3^ The LGK Icon™ consists of 192 sealed sources of Co‐60 arranged in eight sectors with three collimations of 4, 8, and 16 mm. The sources are arranged in a way that creates a radiological focal point (RFP). The positioning system of the unit is the patient couch, which places the target at the center of stereotactic space with submillimeter accuracy. The center of stereotactic space or unit‐center‐point (UCP) coincides with the RFP. The LGK Icon™ system utilizes an on‐board cone beam computed tomography (CBCT) imaging system and an Infrared Intra‐Fraction Motion Management (IFMM) system. The CBCT can be used to align the patient's skull with the reference CBCT image which defines the stereotactic reference. The IFMM system consists of an infrared (IR) camera and an IR reference tool that is fixed to the couch and tracks the intrafractional motion of a reflective marker placed on the patient's nose during treatment delivery. The CBCT along with the IFMM systems enable the use of noninvasive thermoplastic mask instead of conventional frames.^2^, ^3^

Several studies have presented quality assurance (QA) procedures of the LGK Icon™.^4^, ^5^, ^6^, ^7^, ^8^, ^9^ Zeverino *et al*. discussed commissioning procedures of the LGK Icon™.^5^ Knutson et al. described QA techniques for the IFMM system of the LGK Icon™.^6^ AlDahlawi et al. reported the QA procedure for the GK Icon™’s CBCT system.^7^, ^8^ Bhatnagar et al. gave an overview of the tests conducted for the acceptance, commissioning, and periodic QA of an LGK Perfexion™.^9^

The annual QA tests for Gamma Knife^®^ are listed in the vendor’s operation manual, license guidance from the Nuclear Regulatory Commission (NRC), and in title 10 of the code of federal regulations part 35, section 600.^10^, ^11^, ^12^ There are several annual QA tests such as relative output factors (ROF), radiation profile measurements, sector counting, and coincidence of UCP and RFP that are currently being performed using radiochromic or radiographic film.^4^, ^5^, ^9^ Film is a good candidate for small field dosimetry due to its high spatial resolution and availability.^13^ However, there are multiple challenges with using film. First, film does not allow for real‐time measurements and requires a batch‐specific calibration, both of which make film measurements time‐consuming. Additionally, there are several uncertainties associated with film measurements such as handling uncertainties, batch dependency, and uncertainty in calibration procedure. To address these inherent limitations in film dosimetry, the aim of this work is to describe a filmless approach for performing the annual QA of LGK Icon™.

## 2 MATERIALS AND METHODS

The Gamma Knife^®^ annual QA consists of dosimetric, mechanical, and safety components. Dosimetric tests consist of output calibration, ROF, and also radiation profile measurements. Profiles are usually described by their full‐width‐half‐maximum (FWHM) and penumbra for all collimator sizes as compared to the treatment planning system (TPS). Additional tests consist of sector uniformity/sector counting and coincidence of UCP and RFP.

### 2.A Dosimetry

#### 2.A.1 Output calibration

**Fig. 1:**
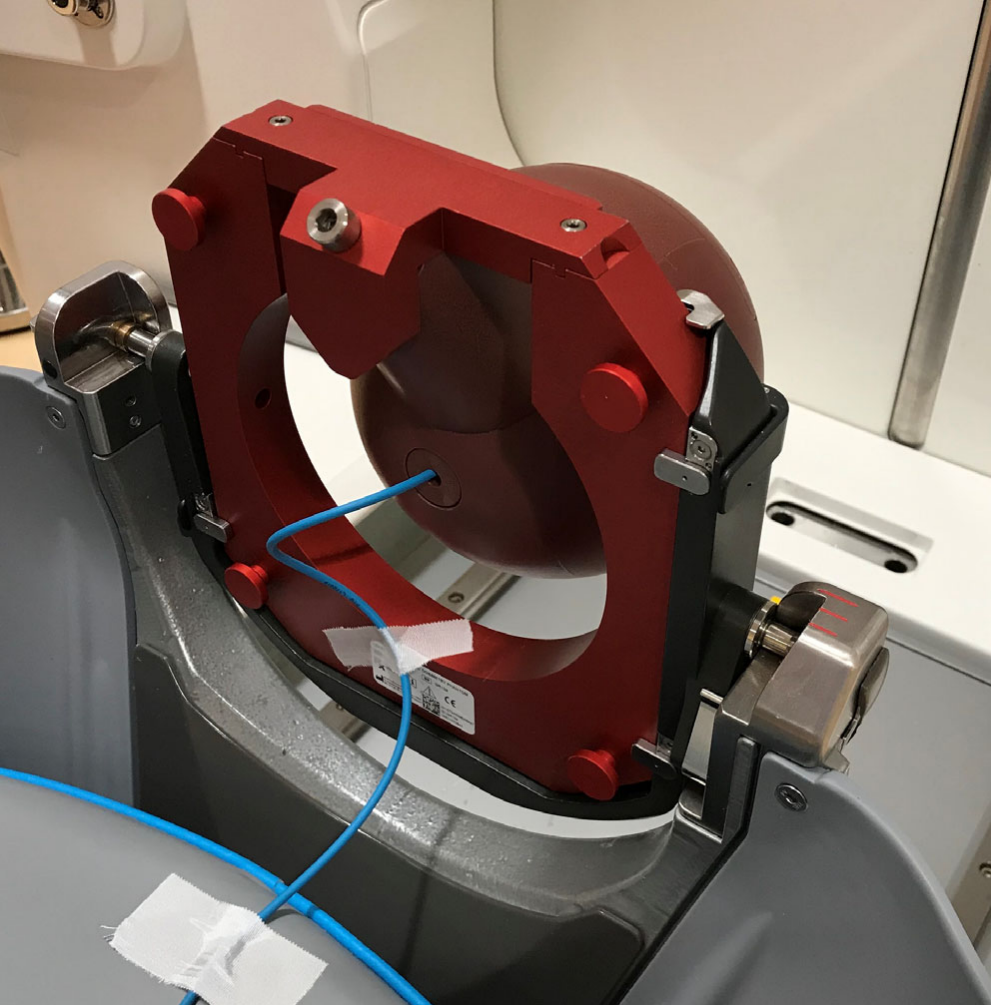
Ionization chamber placed in the 16‐cm diameter Solid Water phantom.

Dosimetric components of the annual QA were performed using Elekta Solid Water 16 cm diameter sphere phantom as shown in Fig. 1. The output calibration was performed according to IAEA TRS 483 protocol using two Accredited Dosimetry Calibration Laboratory (ADCL) calibrated ionization chambers, which included the Capintec (Capintec Inc. Pittsburgh, PA, USA) model PR‐05P 7.6 and PTW (PTW, Freiburg, Germany) model T31010 with PTW Unidos 10005 electrometer (PTW, Freiburg, Germany).^13^ The correction factors in table 14 of the TRS 483 protocol was used for the dose calculation. The dose rate (in Gy/min) was measured for the 16‐mm collimation at the RFP which is at the center of the Solid Water phantom. The measured dose rate was then compared to the output of the TPS.

#### 2.A.2 Relative output factors

ROFs were measured with two methods: (a) using film and (b) a synthetic diamond detector. The results of both measurements were compared to the TPS values. The TPS output factor for 16, 8, and 4 mm collimators are 1, 0.9005, and 0.814, respectively.

**Fig. 2:**
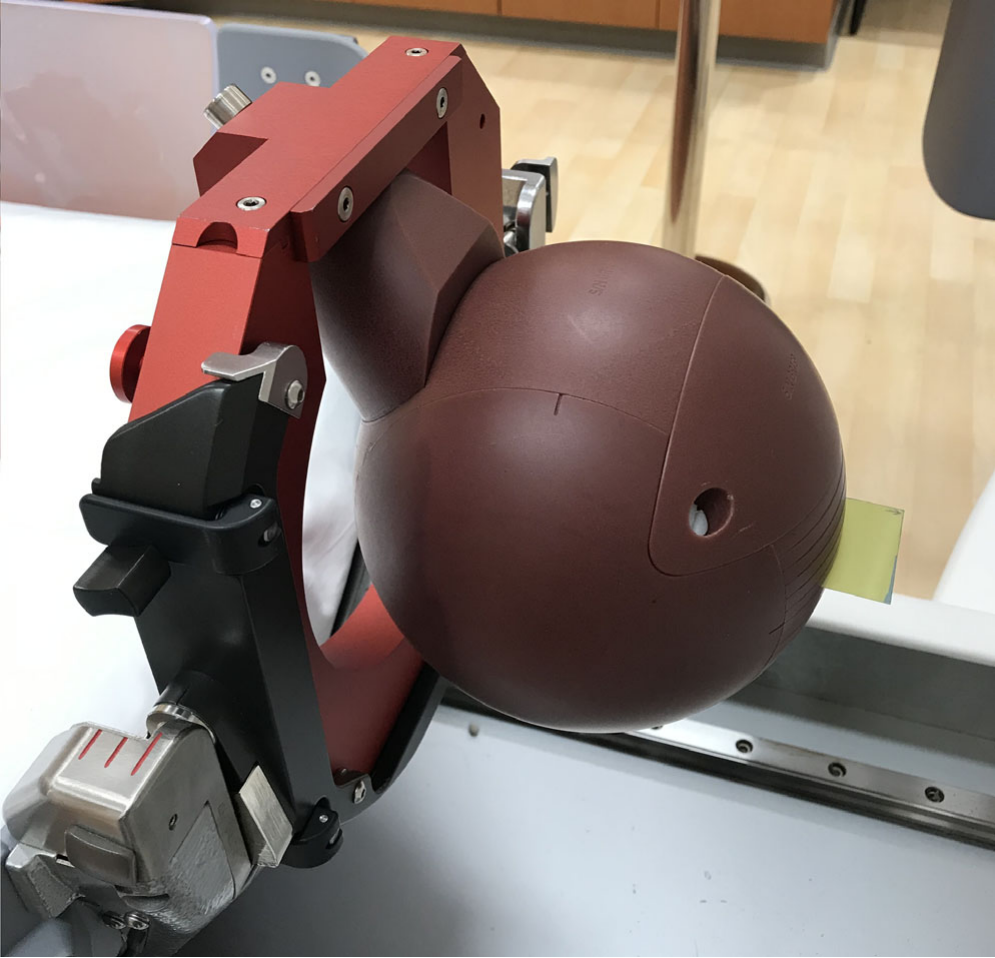
Relative output factors measurement setup using film inside the Solid Water phantom.

Film: The measurements were performed using Gafchromic EBT3 films (Ashland Inc., Bridgewater, New Jersey, USA) in axial (XY) and coronal (XZ) planes placed in the central insert inside the spherical Solid Water phantom (shown in Fig. 2). Three treatment plans were generated for the three collimator settings to deliver the maximum dose of 10 Gy. Therefore, six films were irradiated in total. The films had to be calibrated to convert the intensity levels to absolute dose. In order to create the calibration curve, 14 films from the same batch were irradiated inside the solid water phantom using a 16‐mm collimator to 0.5, 1, 1.5, 2, 3, 4, 5, 6, 7, 8, 9, 10, and 11 and 12 Gy. The films were scanned using an Epson EXPRESSION 10000 XL scanner (Epson America, Inc., Long Beach, California, USA) with 300 dpi resolution, 48‐bit color, and no color correction. After irradiation, there was a 24‐h waiting time prior to scanning. Care was taken to scan each film in the same location to avoid the variation in spatial sensitivity of the scanner. The films were marked on their corner to get irradiated and scanned in the same orientation.^14^, ^15^ All scanned films were analyzed with RIT (Radiological Imaging Technology, Colorado Springs, CO) film dosimetry software in green channel.^16^, ^17^ The calibration curve was built in RIT and applied to the six irradiated films. The region of interest (ROI) was chosen as 1 × 1 mm^2^ to allow reasonable statistics while minimizing volume averaging. The relative output factors were calculated using:

(1) $${\text{OF}}_{\text{measured}}=\frac{D_{x}}{D}\times {\text{OF}}_{\text{TPS}}$$

For every collimator size *x*, D_x_ is the mean measured dose inside a small ROI of an irradiated film, D is the known delivered dose, and OF_TPS_ is the TPS output factor. For example, if 10 Gy was delivered using an 8 mm shot (which has an OF of 0.9005) and 10 Gy was measured, the OF would be 0.9005. If we measure 10.1 Gy then the OF would be 10.1/10 × 0.9005 = 0.9095.

Active detector: The second measurement technique utilized a PTW MicroDiamond detector (T60019). The detector was placed in the Solid Water Phantom along the couch (Z axis) and irradiated for 2 min with all three collimator settings. A correction factor obtained from IAEA TRS 483, table 25 and was applied to our readings ^13^. The PTW MicroDiamond detector was chosen as it has the smallest correction factors (CF) for the 8‐mm (1.005) and 4‐mm (0.993) collimators compared to the other five detectors listed in the table 25 of IAEA TRS 483.^13^

### 2.B Sector uniformity/source counting

Two techniques can be used to ensure that every source is present in the unit.

**Fig. 3:**
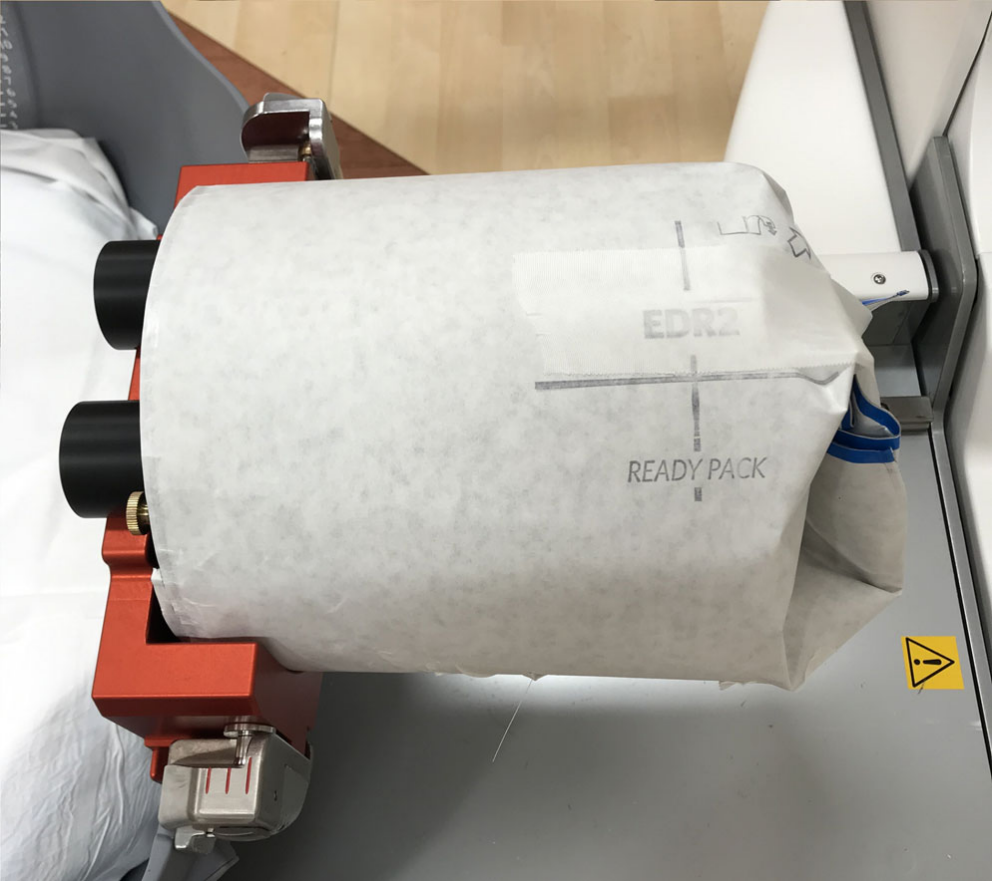
The CATPHAN phantom wrapped in a radiographic film for the source counting measurement.

Film: The first technique can utilize either radiographic or radiochromic film.^18^ As shown in Fig. 3, a CATPHAN phantom (The Phantom laboratory, Salem NY, USA) was wrapped in a radiographic film and placed at the UCP and all sectors were opened to the 4‐mm collimator. After 10 min of exposure, the film was removed and the number of spots (with each spot representing the exposure from an individual source) was manually counted.

Active detector: The second technique is to measure sector uniformity with an active detector. Sector uniformity was measured by placing the PTW 310310 ionization chamber at the UCP and recording the total charge collected when the chamber was exposed to each sector individually. The specified tolerance by the manufacturer is 2% for any given sector. In the event of missing a source, a sector reading difference of about 4% can be expected, since each sector consists of 24 sources with 4.16% contribution from each source if uniformly distributed. However, missing a source from a sector that is +2% in reading will make the sector reading around −2%. Therefore, we recommend performing the sector uniformity/source counting using film if the variation is more than ±1.5%.

### 2.C Radiation profiles

Radiation profiles were obtained using radiographic films and a MicroDiamond detector.

Film: Films were placed inside the spherical phantom in the axial and coronal orientations. Six films were irradiated to a maximum dose of 10 Gy using 4, 8, and 16 mm collimation sizes. Irradiated film were scanned and intensity levels were converted to absolute dose using the calibration curve.

**Fig. 4:**
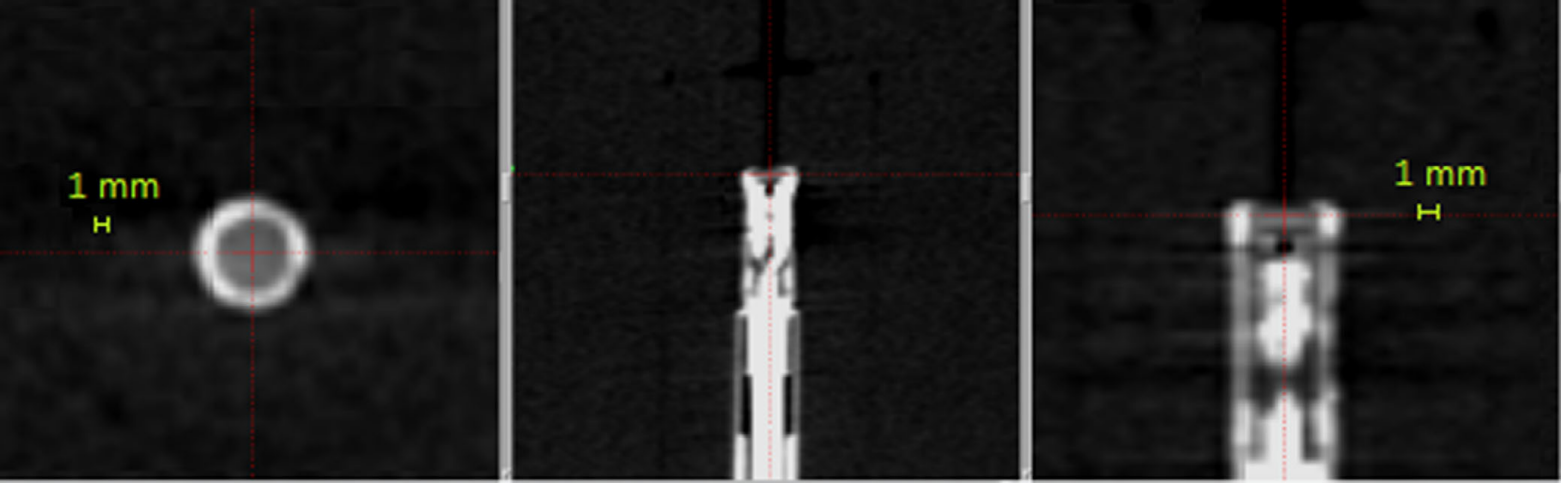
Cone beam computed tomographyCBCT of the MicroDiamond detector and centering it at the unit center point.

Active detector: For the measurement of the beam profiles using a MicroDiamond detector, the detector was placed in the Solid Water phantom and a CBCT was taken. The couch was then shifted to place the center of the detector at center coordinate which coincides with the UCP (100 mm, 100 mm, 100 mm) as shown in Fig. 4.

The spherical phantom with the detector inside was then translated across the radiation center. The couch was manually shifted in service mode with help from the service engineer. The couch was translated along the three orthogonal directions from the X, Y, or Z coordinate of 60–140 mm while the other two coordinates were fixed at UCP coordinate 100 mm. The step sizes were 1 mm in penumbra, 1–2 mm in the field, and 5–10 mm outside the filed depending on dose gradient. The detector was irradiated by each collimator size for 6 s at each increment to reduce the total measurement time.

An extra measurement was also performed in order to find and compare the signal to noise ratio (SNR) with a short and a longer measurement time. The detector inside the Solid Water phantom was irradiated by a 16‐mm shot at center and 4 cm off center for both 6 and 30 s. The measurements were repeated five times for each case to obtain the mean and standard deviation (SD). The SNR was then calculated as SNR = Mean/SD.

**Fig. 5:**
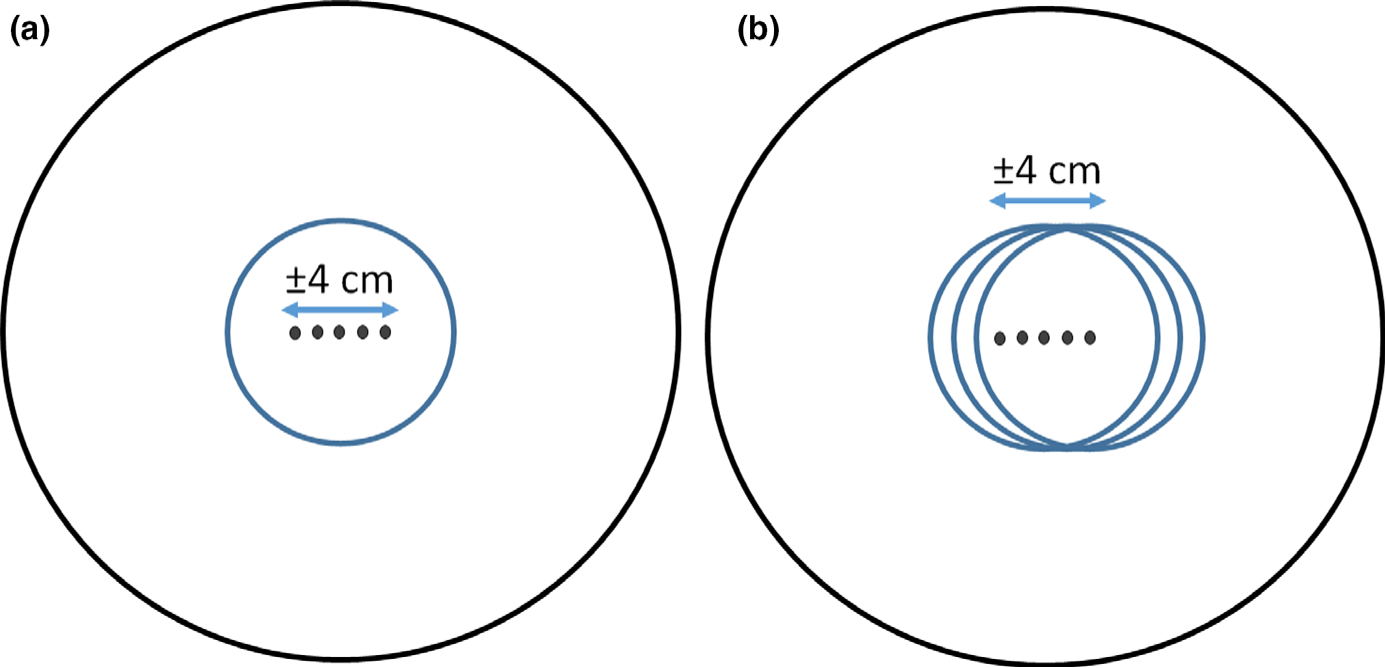
Schematic of the spherical phantom (small blue circle) inside the LGK Icon™ system (large black circle) showing the differences in geometry in (a) treatment planning system vs (b) measurement using MicroDiamond detector. The dots in (a) represent points of measurement while the phantom is stationary and in (b) they represent the detector location which is being translated along with the phantom to obtain the dose profile.

One limitation associated with this method is the difference in the two geometries in the measurement and the TPS. In the measurement geometry the dose profiles are measured by translating the active detector inside the spherical phantom. In the TPS geometry, the profiles are obtained while the phantom is stationary in the center. The point of measurement varies from center of the sphere to 4 cm off center when measuring the dose profile in TPS while the point of measurement is always located at the center of the sphere by translating the detector along with the phantom to measure the profile. A schematic representation of this geometry difference in TPS versus measurement along the X axis is shown in Fig. 5. To measure the path length within the spherical phantom, the geometry in Fig. 5 was simulated in MATLAB (MathWorks, Natick, MA). The spherical phantom was simulated as a circle defined by x=rcosθ and y=rsinθ, where r=8cm and θ=0to2π with increments of π/100. The average distance from the dots (detector locations) to the perimeter of the circle (spherical phantom) was calculated using $d=\sqrt{{\left(x‐i\right)}^{2}+y^{2}}$, where i=0‐4cm with increments of 0.01cm.

Profiles obtained using both techniques were compared against the TPS. The CBCT scan that was acquired for alignment was also used to register to the CT scan of the phantom in order to obtain the radiation profiles in TPS. For each collimator size, a single shot was placed at the detector location inside the spherical phantom. The profiles were then extracted in X, Y, and Z planes from 60 to 140 mm for each shot size (±40 mm from the center).

### 2.D Coincidence of UCP and RFP

**Fig. 6:**
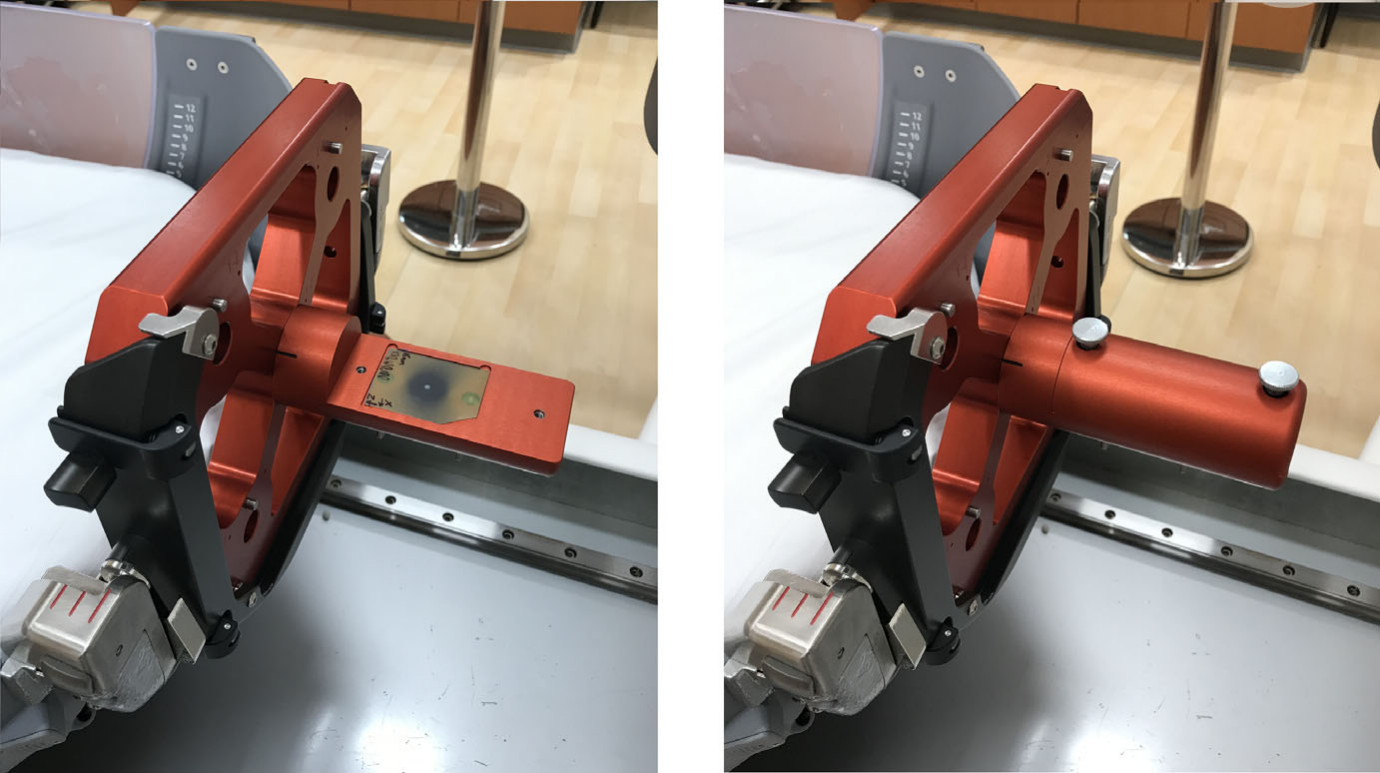
The pinprick film test tool.

Film: The coincidence of UCP and RFP is typically verified using the manufacturer provided pinprick film tool and radiochromic film as shown in Fig. 6. Film is placed in the film holder and a pin punches a hole in the film. This pin point location is at the UCP with coordinates of (100 mm, 100 mm, 100 mm) when the film compartment is at the central location. The film was irradiated in coronal (XZ) and sagittal (YZ) orientations and analyzed using RIT software. The central point of the profile in each direction is measured based on the FWHM of each profile and compared to the pinprick location. The distance between the two points is the difference between UCP and RFP locations.

Active detector: The coincidence of UCP and RFP was also compared to the CBCT imaging isocenter alignment. In this test, a CBCT of the pinprick film tool was taken and the coordinate of the mechanical pointer’s tip was located in the image and compared to the expected coordinates above. The MicroDiamond detector was placed in the Solid Water phantom and centered at the UCP using a CBCT. The coincidence of UCP and RFP was verified by finding the central point of the profile in each direction obtained by the detector. The difference between the central point and 100 mm is the deviation of UCP from RFP measured using the MicroDiamond.

## 3 RESULTS

### 3.A Output calibration

The measured dose rate using the PR‐05P and TN31010 ionization chambers were 2.664 and 2.630 Gy/min. The TPS dose rate at the time of measurement was 2.636 Gy/min. The relative difference between the average of measured dose rate and the TPS value was 0.4%.

### 3.B Relative output factors

The results of measured ROFs for the 8 and 4‐mm collimators using film and the MicroDiamond detector are shown in Table 1. The correction factors for the MicroDiamond detector are shown and were applied to the measured values.

**Table 1:** Relative output factor for the 8 and 4 mm collimation sizes in the treatment planning system (TPS) and measured using radiochromic film and MicroDiamond detector. The last column shows the difference between the two values.

Collimator	ROF in axial plane	ROF in coronal plane	TPS	% Diff
Film				
8 mm	0.919	0.926	0.9005	2.6%
4 mm	0.83	0.83	0.8140	1.9%

Collimator	OF w/o correction	CF	OF with correction	% Diff
MicroDiamond detector				
8 mm	0.892	1.005	0.897	0.3%
4 mm	0.835	0.993	0.829	1.8%

### 3.C Radiation profiles

**Fig. 7:**
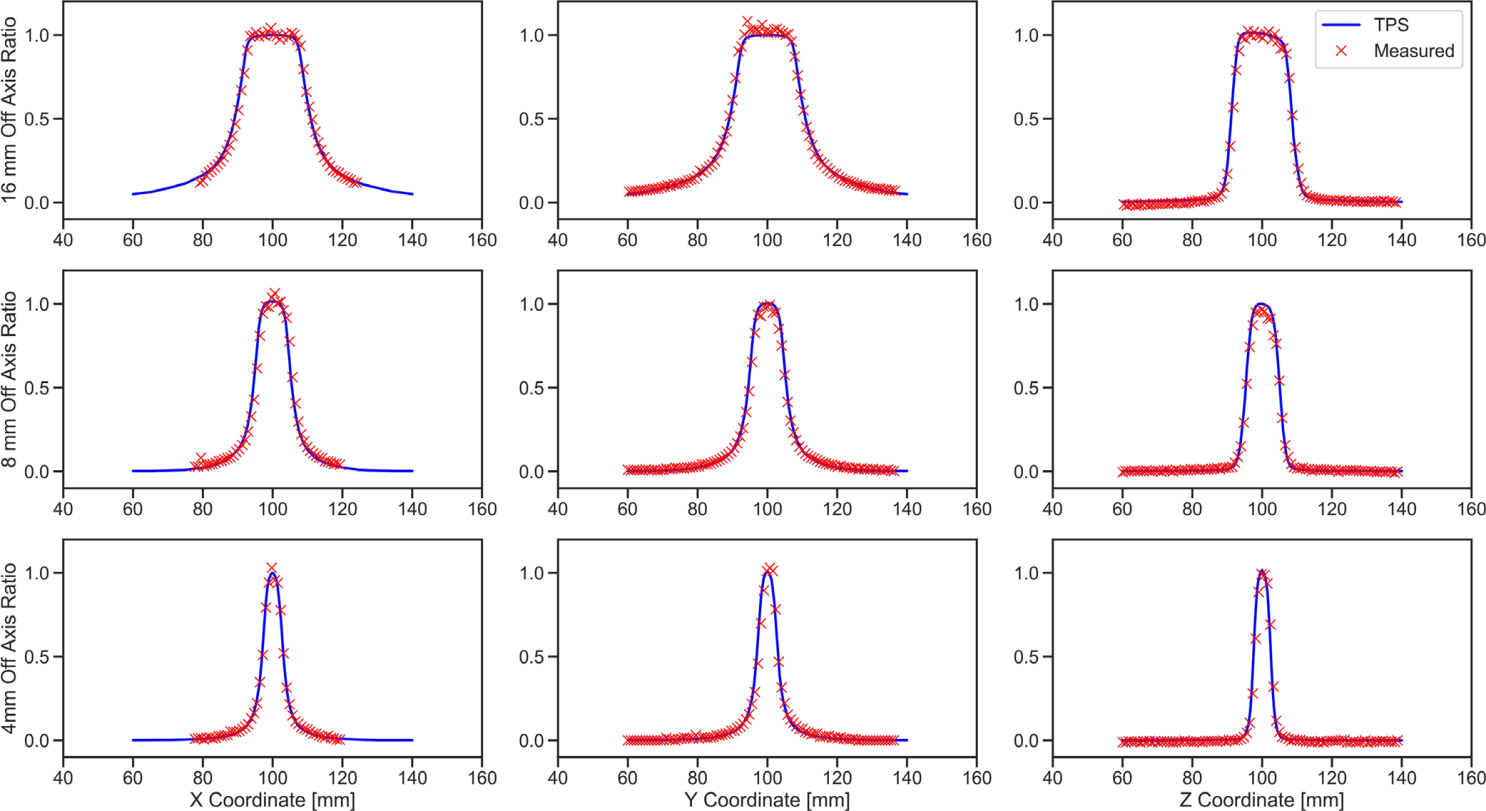
Measured radiation profile along (first column) X, (second column) Y, and (third column) Z for (top row) 16 mm, (middle row) 8 mm, and (bottom row) 4 mm collimators using radiochromic films.

**Fig. 8:**
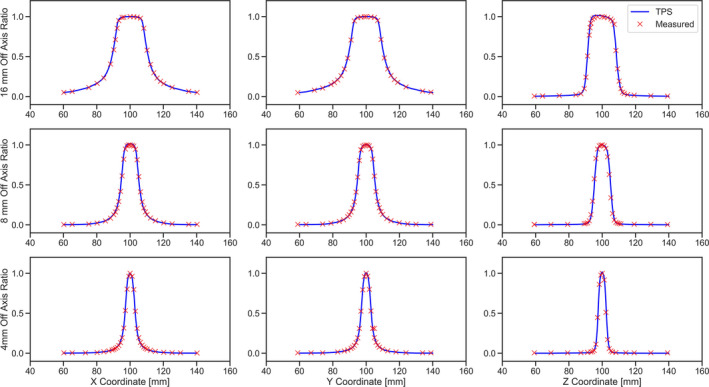
Measured radiation profile along (first column) X, (second column) Y, and (third column) Z for (top row) 16 mm, (middle row) 8 mm, and (bottom row) 4 mm collimators using the MicroDiamond detector.

Figures 7 and 8 show the measured dose profiles along axial, coronal, and longitudinal directions for 4, 8, and 16 mm collimator sizes using film and the MicroDiamond detector, respectively. The radiation profiles for the film measurements were extracted from irradiated films in coronal, sagittal, and axial planes. Tables 2 represents the FWHM and penumbra calculated from the dose profiles obtained using films and MicroDiamond detector. Table 3 shows the calculated SNR at the UCP and 4 cm away from the UCP for both 6 and 30 s measurements.

**Table 2:** Penumbra and full‐width‐half‐maximum (FWHM) for different collimator sizes measured using film and MicroDiamond detector and compared to treatment planning system (TPS) values. The first number in the table represents penumbra and the second number represents FWHM.

Col. size	Profile	Meas. Pen., FWHM [mm]	TPS Pen., FWHM [mm]	Meas. vs TPS Pen., FWHM [mm]
Film				
4 mm	X	2.7, 6.0	2.9, 6.2	0.2, 0.2
	Y	2.9, 5.5	2.9, 6.2	0.0, 0.7
	Z	1.5, 4.9	1.6, 5.0	0.1, 0.1
8 mm	X	3.9, 10.8	4.0, 11.0	0.1, 0.2
	Y	4.2, 10.5	4.1, 11.0	0.1, 0.5
	Z	2.4, 9.7	2.4, 9.8	0.0, 0.1
16 mm	X	9, 21.6	9.2, 21.8	0.2, 0.2
	Y	8.6, 21.5	9.3, 21.8	0.7, 0.3
	Z	2.6, 17.3	2.7, 17.5	0.1, 0.1
MicroDiamond detector				
4 mm	X	2.9, 6.3	2.9, 6.2	0.0, 0.1
	Y	2.9, 6.2	2.9, 6.2	0.0, 0.0
	Z	1.6, 4.9	1.6, 5.0	0.0, 0.1
8 mm	X	4.1, 11.1	4.0, 11.0	0.1, 0.1
	Y	4.2, 11.1	4.1, 11.0	0.1, 0.1
	Z	2.4, 9.7	2.4, 9.8	0.0, 0.1
16 mm	X	9.3, 21.8	9.2, 21.8	0.1, 0.0
	Y	9.3, 21.7	9.3, 21.8	0.0, 0.1
	Z	2.6, 17.4	2.7, 17.5	0.1, 0.1

**Table 3:** Calculated signal to noise ratio at center and 4 cm off center for both 6 and 30 s measurements.

Location	Time [s]	Rdg 1 [nC]	Rdg 2 [nC]	Rdg 3 [nC]	Rdg 4 [nC]	Rdg 5 [nC]	SNR	1/SNR × 100
Center	6	0.3085	0.3085	0.3090	0.3085	0.3085	690.3	0.14
	30	1.544	1.545	1.544	1.543	1.544	2183.5	0.05
Off center	6	0.0148	0.0148	0.0148	0.0149	0.0148	331.4	0.3
	30	0.0737	0.0737	0.0739	0.0737	0.0736	700.7	0.14

**Fig. 9:**
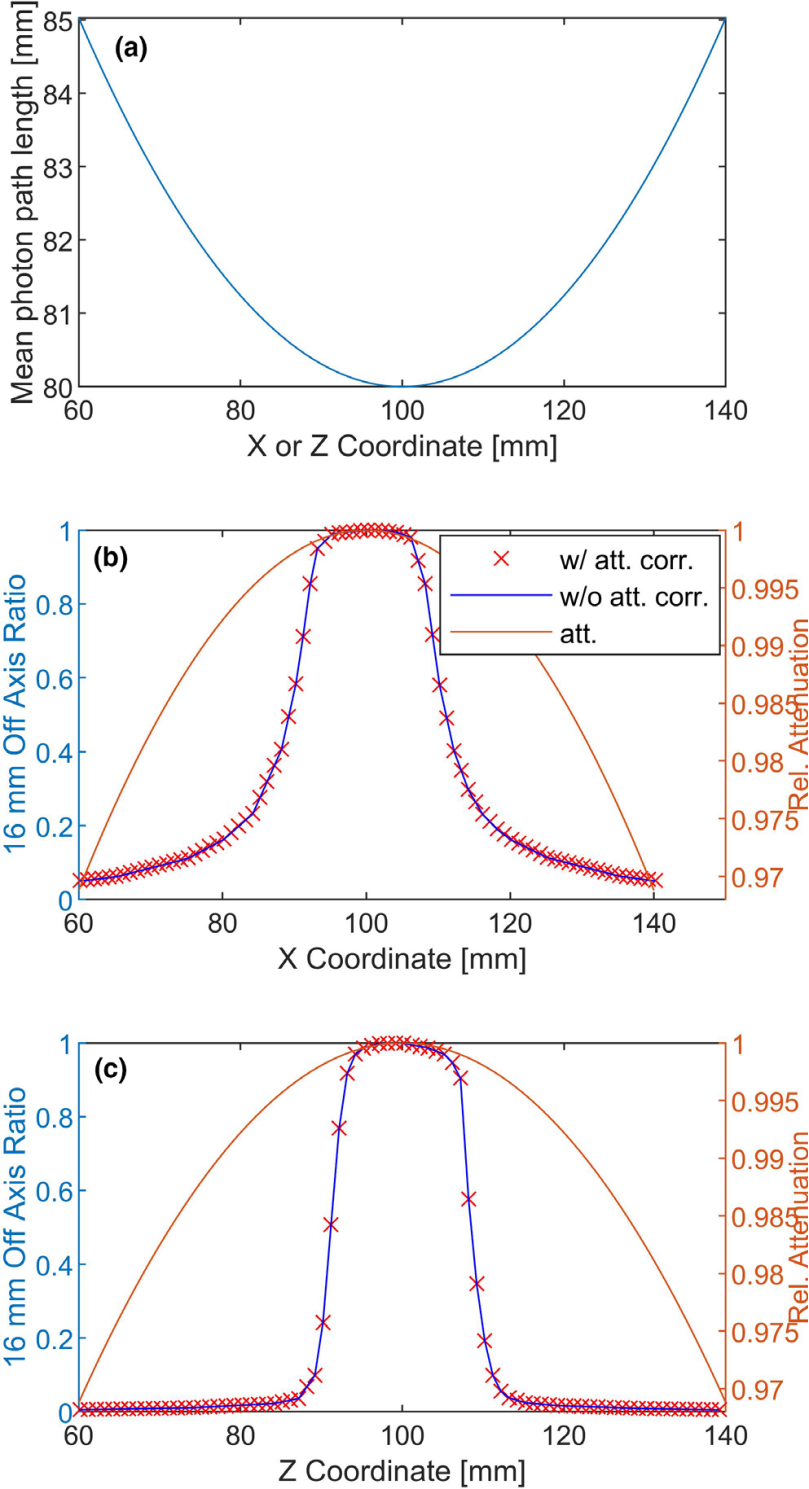
(a) Mean photon path length within the spherical phantom and (b) change in relative attenuation as the point of measurement changes from the center to 4 cm off center. (b) Dose profile of a 16‐mm shot along the X and Z axes using MicroDiamond detector with and without taking in to account the effect of attention (att. corr.: attenuation correction).

The average photon path length within the spherical phantom when the point of measurement changes from the center to 4 cm off center increases from 8 to 8.5 cm [shown in Fig. 9(a)]. This increase in path length causes approximately a 3.1% increase in attenuation at 4 cm off center [shown in Fig. 9(b)].^19^ The dose profile for a collimator size of 16 mm along the X and Z axes along with the correction for photon attention are shown in Figs. 9(b) and 9(c). The effect of extra attenuation on the profile is negligible because the off axis ratio drops with a high gradient with distance from the center.

### 3.D Sector uniformity/source counting

The results for the sector uniformity measured using the PTW ionization chamber is presented in Table 4. The average value for all the sectors were obtained and variation of each sector from the average value is shown. For the source counting using film, the film was removed after 10 min of exposure and the number of spots (with each spot representing the exposure from an individual source) was counted.

**Table 4:** Measured ion chamber reading exposed to each sector with variation of each sector reading from the average value of all readings.

Sector	Reading	Variation (%)
1.000	2.271	−0.34
2.000	2.292	0.58
3.000	2.267	−0.51
4.000	2.285	0.27
5.000	2.285	0.27
6.000	2.285	0.27
7.000	2.265	−0.6
8.000	2.28	0.1

### 3.E Coincidence of UCP and RFP

The difference between the UCP and RFP locations measured using the pinprick film test and MicroDiamond detector in axial, coronal, and sagittal planes are shown in Table 5. The total difference between the UCP and RFP is represented by $\Delta r=\sqrt{X^{2}+Y^{2}+Z^{2}}$.

**Table 5:** The difference between unit center point and radiological focal point measured using film and MicroDiamond detector in X, Y, and Z directions.

Collimator size [mm]	X [mm]	Y [mm]	Z [mm]	∆r [mm]
Film				
4	0.07	0.03	0.28	0.29
8	0.1	0.2	0.21	0.3
16	0.09	0.18	0.2	0.28
MicroDiamond				
4	0.18	0.10	0.21	0.29
8	0.16	0.13	0.23	0.3
16	0.21	0.06	0.15	0.26

## 4 DISCUSSION

Annual QA tests for an LGK Icon™ system were performed using film‐based and film‐less methods. The absolute dose rate for the 16‐mm collimator size was measured using two ADCL ionization chambers. The measured dose rate was within 0.4% of the TPS value.

Sector uniformity was found to be within 0.6% for all eight sectors measured using an ionization chamber. Source counting was performed using radiographic film and the presence of all 192 sources were confirmed. Verifying the presence of all sources was simply performed using ion chamber measurements in a few minutes with the same setup as the dose rate measurement, whereas the radiographic film had to be processed and 192 irradiated spots needed to be counted.

Radiation dose profiles were measured using EBT3 film and a MicroDiamond detector. Excellent agreement was found between TPS and measured dose profiles using the MicroDiamond detector which was within 1%/1 mm vs 2%/1 mm for film. The difference between measured and TPS values of FWHM and penumbra was up to 0.1 mm using the MicroDiamond and 0.7 mm using film. Measuring the radiation profiles using an active detector has several advantages over film. Film measurements take time and there are multiple uncertainties associated with them. The films should be scanned after 24 h and require creating calibration curve which includes irradiating, scanning and analyzing multiple extra films (15 films in this case).^20^ Some of the uncertainties associated with film measurements include handling uncertainties, batch dependency, variation in response uniformity and reproducibility, uncertainty in calibration procedure, and scanning uncertainties.^16^, ^19^ On the other hand, dose profiles can be measured with a simpler method in a shorter period of time with lower uncertainty using the MicroDiamond detector. For the active detector measurement, the profiles can be obtained only by translating the detector across the radiation field using the couch. Using this method, the total measurement time was less than 2 h. SNR measurements at center and 4 cm off center showed acceptable results at 6 s compared to those at 30 s. Six seconds was the shortest available collecting time using the Unidos 10005 electrometer.

The MicroDiamond detector has an active volume of 0.004 mm^3^ with a thickness of 0.001 mm and an area of 4 mm^2^. The TPS has a resolution of 0.5 mm × 0.5 mm × 0.5 mm (0.125 mm^3^). Therefore, active volume of the detector is much smaller than the TPS voxel size. CBCT isocenter alignment was tested using the Elekta Daily QA tool. This was tracked throughout the year and did not deviate more than 0.2 mm from baseline at installation. Furthermore the CBCT precision test was done using the pinprick tool at the time of annual QA and the result was within 0.2 mm. With the couch having an uncertainty of <0.3 mm, the overall uncertainty in CBCT isocenter alignment with the RFP was <0.5 mm.

ROFs were measured using EBT3 film and a MicroDiamond detector. The ROF for the 8 and 4‐mm collimators were found to be 0.4% and 1.8% different from TPS values using the MicroDiamond detector versus 2.6% and 1.9% for film, respectively. The size of ROI impacts the measured ROF for film measurement. Reducing the size of ROI will increase the statistical uncertainty while increasing it increases volume averaging. The main cause of discrepancy in the active detector could be volume averaging effect due to the size of the detector and small field size. The results indicate that the ROF can be measured more closely to the TPS values using the MicroDiamond detector compared to film measurement. Additionally, the ROF measurement using the active detector was performed in a much shorter time. With the active detector, only three output measurements were performed and the results were obtained using correction factors. Conversely, with the films, six films had to be irradiated, scanned, and analyzed after 24 h of irradiation and took about 3 h.

Verification of UCP and RFP coincidence using pinprick film test showed 0.29, 0.3, and 0.28 mm variation for 4, 8, and 16 mm collimations using film. Using the MicroDiamond detector, the variations between UCP and RFP were 0.29, 0.3, and 0.26 mm for the 4, 8, and 16 mm collimator sizes, respectively. It should be noted that no extra measurement was needed to perform the UCP and RFP coincidence test when using the MicroDiamond detector since the dose profiles were used to extract the results, which results in additional time saved.

## 5 CONCLUSION

The measured output calibration using two ADCL calibrated ionization chambers was in agreement (within 0.4%) with the TPS. ROFs were measured with film and a synthetic diamond detector, and a closer agreement to the TPS values was obtained using the synthetic diamond detector. The ROF for the 8 and 4‐mm collimators were 0.4% and 1.8% different from TPS values using the MicroDiamond detector versus 2.6% and 1.9% for film, respectively. Comparable results were obtained by measuring the dose profiles using film and a synthetic diamond detector. The agreement between the measured dose profiles using the MicroDiamond detector and film with the TPS were within 1%/1 mm and 2%/1 mm, respectively. Sector uniformity/sector counting was successfully performed using radiographic film and an ionization chamber. Verification of UCP and RFP coincidence using the MicroDiamond detector yielded similar results compared to the pinprick film test. The variation between UCP and RFP for 4, 8, and 16 mm collimations were 0.29, 0.3, and 0.28 mm using pinprick film and 0.29, 0.3, and 0.26 mm using the MicroDiamond detector, respectively. Therefore, the findings of this study show the feasibility of using filmless techniques for the annual QA of an LGK Icon™ system. Utilizing active detector‐based QA simplifies the procedure and saves time without loss of accuracy.

## AUTHORS’ CONTRIBUTIONS

Borna Maraghechi was involved with the measurements and writing the manuscript. Taeho Kim was involved with the measurements and proofreading the manuscript. Timothy J. Mitchell was involved with proofreading and editing the manuscript, S. Murty Goddu was involved with proofreading and editing the manuscript, Joe Dise was involved with proofreading and editing the manuscript, James A. Kavanaugh was involved with proofreading and editing the manuscript, Jacqueline E. Zoberi was involved with proofreading and editing the manuscript, Sasa Mutic was involved with proofreading and editing the manuscript. Nels C. Knutson was involved with the measurements, proofreading the manuscript, and overall design and guidance of the work. All authors read and approved the manuscript.
